# A systematic analysis of IBD GWAS loci identifies most probable causal genes impacting intestinal epithelial functions

**DOI:** 10.64898/2026.07.30.26359349

**Published:** 2026-08-02

**Authors:** Isabelle Hébert-Milette, Virginie Mercier, Jean Paquette, Gabrielle Boucher, Chloé Lévesque, Philippe Goyette, John D. Rioux

## Abstract

**Background:**

Genome-wide association studies have identified >200 loci associated with IBD, yet the causal gene for most remains unknown. As multiple epithelial functions have been linked with susceptibility to IBD, there is a need to prioritize candidate causal genes for functional studies in this cellular context.

**Methods:**

Using a standardized definition of regions implicated by index SNPs from three GWAS studies, we categorized regions as containing: (1) a known casual gene, (2) a single gene or (3) multiple genes. We then developed an *IBD Priority Score* to rank genes based on genetic, genomic and functional data. We next developed and applied an *Epithelial Priority Score* , based on expression patterns and quantitative traits, to prioritize genes for functional validation in epithelial models. Two candidate genes identified through this approach were tested for their impact on viral response pathways in HT-29 cells.

**Results:**

The *IBD Priority Score* prioritized a single gene in 71 of the 104 regions containing multiple genes. The *Epithelial Priority Score* identified 31 epithelial candidates. Functional studies demonstrated that IRF6 enhanced, whereas IRF8 suppressed, antiviral responses in intestinal epithelial cells stimulated with Poly(I:C).

**Conclusions:**

Combining multiple genetic, genomic, and functional data is a useful approach for prioritizing the most likely causal gene within IBD GWAS loci, and for prioritizing functional validation studies in epithelial cells and tissues. Moreover, we provide functional evidence for two IBD genes playing a role in the regulation of anti-viral responses in intestinal epithelial cells.

**Lay Summary:**

Genetic studies have played an important role in identifying disease pathways. Cells lining the gut, epithelial cells, play an important role in IBD. The current study provides an approach for identifying key IBD pathways in these cells.

**Key Messages:**

**What is already known?**

GWAS have identified more than 200 IBD susceptibility loci, but the causal genes remain unknown for most regions, and growing evidence implicates intestinal epithelial dysfunction in the development of IBD.

**What is new here?**

We developed an approach that prioritizes likely causal genes within IBD loci and functionally demonstrated that two of these (IRF6 and IRF8) are important for their role in antiviral pathways in human intestinal epithelial cells.

**How can this study help patient care?**

Speeding up the discovery of genes and functions relevant to IBD susceptibility pathways in intestinal epithelial cells will accelerate the discovery of novel therapies that are complementary to existing advanced therapies that target genes and functions in immune cells.

## Full Text Availability

The license terms selected by the author(s) for this preprint version do not permit archiving in PMC. The full text is available from the preprint server.

